# Stromal-Immune Score-Based Gene Signature: A Prognosis Stratification Tool in Gastric Cancer

**DOI:** 10.3389/fonc.2019.01212

**Published:** 2019-11-12

**Authors:** Hao Wang, Xiaosheng Wu, Yiming Chen

**Affiliations:** ^1^First Department of Gastrointestinal Surgery, Hainan General Hospital, Hainan Medical University, Haikou, China; ^2^Guangdong Provincial Key Laboratory of Gastroenterology, Department of Gastroenterology, Nanfang Hospital, Southern Medical University, Guangzhou, China

**Keywords:** gastric cancer, microenvironment, stromal, immune, prognosis, prediction

## Abstract

**Background:** A growing amount of evidence has suggested the clinical importance of stromal and immune cells in the gastric cancer microenvironment. However, reliable prognostic signatures based on assessments of stromal and immune components have not been well-established. This study aimed to develop a stromal-immune score-based gene signature in gastric cancer.

**Methods:** Stromal and immune scores were estimated from transcriptomic profiles of a gastric cancer cohort from TCGA using the ESTIMATE algorithm. A robust partial likelihood-based Cox proportional hazard regression model was applied to select prognostic genes and to construct a stromal-immune score-based gene signature. Two independent datasets from GEO were used for external validation.

**Results:** Favorable overall survivals were found in patients with high stromal score (*p* = 0.014) and immune score (*p* = 0.045). Forty-five stromal-immune score-related differentially expressed genes were identified. Using a robust partial likelihood-based Cox proportional hazard regression model, a gene signature containing SOX9, LRRC32, CECR1, and MS4A4A was identified to develop a risk stratification model. Multivariate analysis revealed that the stromal-immune risk score was an independent prognostic factor (*p* = 0.018). Based on the risk stratification model, the cohort was classified into three groups yielding incremental survival outcomes (log-rank test *p* = 0.0004). A nomogram integrating the risk stratification model and clinicopathologic factors was developed. Calibration and decision curves showed a better performance and net benefits for the nomogram. Similar findings were validated in two independent cohorts.

**Conclusion:** The stromal-immune score-based gene signature represents a prognosis stratification tool in gastric cancer.

## Introduction

Gastric cancer is one of the leading causes of cancer-related mortality, and it characteristically has widely varying prognostic outcomes (1). The stage, determined according to the tumor, node, and metastasis (TNM) system, has been generally considered one of the main tools for routine prognostication for gastric cancer in treatment practice (2). However, wide variation in prognostic outcomes has been reported for gastric cancer patients with the same TNM stages and similar clinical management approaches (3–5), indicating that the current TNM stage system provides incomplete information for prognosis stratification of gastric cancer. Thus, new strategies are warranted to improve prognosis stratification and survival outcome prediction over the current staging system.

As one of the malignant solid tumors, gastric cancer tissue consists of not only tumor cells but also tumor-related stromal cells, infiltrating immune cells, and other normal epithelial cells. Stromal cells are increasingly thought to play important roles in tumor growth, disease progress, and drug resistance (6–8); meanwhile, infiltrating immune cells act in a context-dependent manner, associated with tumor invasion and metastasis (9, 10). An increasing amount of evidence has suggested the clinical importance of stromal cells and immune cells in the microenvironment of gastric cancer tissues (11–16). Therefore, incorporating a prognostic repertoire of stromal cells and immune cells into the current TNM staging system could help to improve the prognosis stratification of gastric cancer patients.

ESTIMATE (Estimation of STromal and Immune cells in MAlignant Tumor tissues using Expression data) is a newly developed algorithm that takes advantage of the unique properties of the transcriptional profiles of cancer tissues to infer tumor cellularity as well as the different infiltrating normal cells (17). The algorithm imputes stromal and immune scores to predict the level of infiltrating stromal and immune cells based on specific gene expression signatures of stromal and immune cells. In the present study, the ESTIMATE algorithm was applied to estimate the stromal and immune scores of a series of gastric cancer tissues based on their transcriptional profiles, and a stromal-immune score-based gene signature was subsequently developed for prognosis stratification in gastric cancer.

## Methods

### Data Preparation and Estimation of Stromal and Immune Scores

The Cancer Genome Atlas (TCGA) level 3 gene expression data of gastric cancer tissues were obtained from the Genomic Data Commons (GDC, available at: https://portal.gdc.cancer.gov/) Data Portal on Mar 7, 2019. The expression profiles for tumors with “Stomach” as the primary site and the disease types of “Adenomas and Adenocarcinomas” or “Cystic, Mucinous and Serous Neoplasms” from a “TCGA-STAD” project were included. Clinicopathologic data for the corresponding patients, including gender, race, age, tumor location, histology classification, differentiation grade, tumor stage, and survival information, were also retrieved from the database. Only patients with both survival information and expression data available at that time point were included in this study. Fragments per kilobase of exon per million reads mapped (FPKM) were used for expression quantification for a total of 19,745 protein-coding genes that have been annotated in the TCGA data portal. The ESTIMATE algorithm was applied to the normalized expression matrix for estimating the stromal and immune scores for each gastric cancer sample (17). Two independent datasets from the Gene Expression Omnibus (GEO) database were used for external validation in this study, including 433 gastric cancer patients from Series GSE84437 and 300 patients from Series GSE62254. For all patients from the GEO database, a normalized expression matrix was used directly for the analyses. Access to the de-identified linked dataset was obtained from TCGA and GEO in accordance with the database policy. For analyses of de-identified data from the TCGA and GEO databases, institutional review board approval and informed consent were not required.

### Correlations Between Prognoses and Stromal/Immune Scores

Overall survival was used as the primary prognosis endpoint and was estimated by the Kaplan-Meier survival estimator. Based on the stromal and immune scores estimated from each gastric cancer sample, corresponding patients were classified into two groups, and prognoses for each group were examined. To identify the best score cutoff for grouping patients most significantly, a previously published method, maximally selected rank statistics, was employed for optimal cutoff identification by using the R package “maxstat” (18). The survival outcomes of the two groups were compared by log-rank tests.

### Differentially Expressed Gene (DEG) Identification

Through the optimal score cutoff mentioned above, patients were divided into two groups, namely the high stromal (or immune) score group and the low stromal (or immune) score group. Linear models were used to identify DEGs between the two groups (high-score group vs. low-score group) by using an R package “limma” (19). A false discovery rate (FDR) adjusted *p*-value <0.0001 combined with a simultaneously absolute value of log_2_ (fold change) >4 was set as the threshold for DEG identification. Genes that were overexpressed in the high-score group compared with the low-score group were considered “overexpressed DEGs” and those that were underexpressed in the high-score group were considered “underexpressed DEGs.” The expression patterns of significant DEGs were visualized on a heatmap with unsupervised hierarchical clustering analyses using the complete linkage method to measure distances between clusters.

### Gene Ontology and KEGG Pathway Enrichment Analyses

Enrichment analyses of Gene Ontology terms, including cellular component, molecular function, and biological process, and of the KEGG pathway were performed for all DEGs shared in the stromal score groups and the immune score groups. An FDR adjusted *p*-value <0.05 was considered as statistically significant for Gene Ontology and KEGG pathway over-representation tests.

### Stromal-Immune Score-Based Gene Signature and Risk Stratification Model

Genes with the lowest log-rank *p*-value <0.01 for survival comparison at the optimal expression cutoffs were considered as prognostic genes. Prognostic gene identification was performed for all DEGs shared in both the stromal score groups and the immune score groups, whereby overexpressed and underexpressed prognostic DEGs were obtained, respectively. Among all prognostic DEGs, a robust partial likelihood-based Cox proportional hazard regression survival model was applied to select prognostic signature genes (20). A 3-fold cross-validation (sample size, training set: validation set = 2:1) and 1,000 iterations was conducted to reduce the potential instability of the results. A forward selection was employed to generate a series of gene signatures, and the optimal gene signature was identified based on the statistics of negative log-likelihood and Akaike Information Criterion (AIC). The optimal gene signatures in this analysis were further compared with two previously reported prognostic gene signatures. One was the CXCR family, of which three members, CXCR4, CXCR6, and CXCR7, were reported as a prognostic signature in gastric cancer (21). Another was a cell cycle-related signature, containing MARCKS, CCNF, MAPK14, INCENP, and CHAF1A (22). The risk stratification values of the above signatures were compared by the −2log likelihood statistic in Cox regression analysis. A smaller value indicated a better ability to predict and stratify survival outcome. Based on the optimal prognostic signature genes identified in the current analysis, a specific model was developed for risk stratification: (i) calculate the score for each signature gene; for a favorable prognostic gene, 0 for overexpression and 1 for underexpression; for an unfavorable prognostic gene, 1 for overexpression and 0 for underexpression; (ii) calculate the total risk score, namely sum up all of the scores for all signature genes; (iii) stratify into risk group based on the level of the total risk score; if risk score = 0, classify into the low-risk group; if risk score = 1, classify into the moderate-risk group; if risk score ≥2, classify into the high-risk group.

### Statistical Analyses

All analyses were performed with R version 3.4.1 (http://www.R-project.org) and its appropriate packages. Stromal and immune scores were calculated by using the “estimate” package with default parameters. The robust likelihood-based survival modeling was performed by using the “rbsurv” package with 3-fold cross-validation and 1,000 iterations. Three packages, namely “GOstats,” “Category,” and “KEGG.db,” were used for GO and KEGG enrichment analyses. Heatmaps and Venn diagrams were constructed by using the “pheatmap” and “VennDiagram” packages, respectively. Data were analyzed with standard statistical tests as appropriate. Multiple testing was adjusted by using the FDR method. For identifying independent risk factors for overall survival, multivariate Cox regression analysis was performed to adjust covariates.

## Results

### Estimation of Infiltrating Cells and Stromal and Immune Scores

A cohort containing 354 gastric cancer patients with available expression data and clinical information in TCGA database was analyzed. The clinicopathological characteristics of the analyzed patients are listed in Table 1. Among them, 125 (35.3%) patients were female, and the majority had tumors located in the body (35.0%) or distal (37.3%) stomach. The tumor stage upon presentation was stage I in 14.1%, stage II in 33.1%, stage III in 41.5%, and stage IV in 10.2% of cases. The infiltrating cells and tumor purity of the tumor tissue were estimated by incorporating two gene signatures in the ESTIMATE algorithm. A “stromal signature” was designed to capture the presence of stromal cells in tumor tissue, and an “immune signature” was aimed to represent the infiltration of immune cells in tumor tissue. Each signature used for the current estimation contained all of the 141 genes proposed (Supplementary Table 1). Based on these two signatures, stromal scores and immune scores were generated to reflect the presence of stromal and immune cells, respectively, and these were combined to represent a measurement of tumor purity by using single-sample gene set-enrichment analysis (ssGSEA) (23, 24). The stromal score for the analyzed cohort ranged from −1919.07 to 2064.31, and immune score was distributed between −1112.53 and 3168.56; in general, patients yielded a higher immune score than stromal score in the entire cohort (Supplementary Figure 1).

**Table 1:** Clinicopathologic characteristics of patients in different risk groups.

**Characteristics**	**Whole cohort (*n* = 354)**	**Log-rank *p***	**Low risk (*n* = 42)**	**Moderate risk (*n* = 156)**	**High risk (*n* = 156)**	***p***
Gender		0.117				0.781
Female	125 (35.3)		17 (40.5)	56 (35.9)	52 (33.3)	
Male	229 (64.7)		25 (59.5)	100 (64.1)	104 (66.7)	
Race^†^		0.079				0.229
Asian	73 (20.6)		10 (28.6)	38 (27.1)	25 (18.7)	
Black	12 (3.4)		2 (5.7)	7 (5.0)	3 (2.2)	
White	224 (63.3)		23 (65.7)	95 (67.9)	106 (79.1)	
Age		0.148				0.394
<70 years	213 (60.2)		22 (52.4)	92 (59.0)	99 (63.5)	
≥70 years	141 (39.8)		20 (47.6)	64 (41.0)	57 (36.5)	
Tumor location^†^		0.555				0.708
Proximal	45 (12.7)		5 (12.2)	19 (12.6)	21 (14.2)	
Body	124 (35.0)		13 (31.7)	54 (35.8)	57 (38.5)	
Distal	132 (37.3)		18 (43.9)	56 (37.1)	58 (39.2)	
EGJ	39 (11.0)		5 (12.2)	22 (14.6)	12 (8.1)	
Histology classification		0.057				0.002
Signet ring cell	11 (3.1)		0 (0.0)	4 (2.6)	7 (4.5)	
Diffuse type	61 (17.2)		5 (11.9)	17 (10.9)	39 (25.0)	
Intestinal type	162 (45.8)		25 (59.5)	85 (54.5)	52 (33.3)	
Others	120 (33.9)		12 (28.6)	50 (32.1)	58 (37.2)	
Differentiation grade		0.295				0.001
G1	9 (2.5)		2 (4.8)	3 (1.9)	4 (2.6)	
G2	128 (36.2)		24 (57.1)	68 (43.6)	36 (23.1)	
G3	208 (58.8)		14 (33.3)	82 (52.0)	112 (71.8)	
Gx	9 (2.5)		2 (4.8)	3 (1.9)	4 (2.6)	
Tumor stage^†^		<0.001				0.044
I	50 (14.1)		12 (28.6)	24 (15.4)	14 (9.2)	
II	117 (33.1)		10 (23.8)	56 (35.9)	51 (33.6)	
III	147 (41.5)		16 (38.1)	64 (41.0)	67 (44.1)	
IV	36 (10.2)		4 (9.5)	12 (7.7)	20 (13.2)	
Stromal score		0.014				<0.001
Low	127 (35.9)		38 (90.5)	67 (42.9)	22 (14.1)	
High	227 (64.1)		4 (9.5)	89 (57.1)	134 (85.9)	
Immune score		0.045				<0.001
Low	58 (16.4)		32 (76.2)	20 (12.8)	6 (3.8)	
High	296 (83.6)		10 (23.8)	136 (87.2)	150 (96.2)	

† *Patients with information unavailable on race (45 patients, 12.7%), tumor location (14 patients, 40%), and tumor stage (4 patients, 1.1%) were excluded from the comparison*.

### Association of Stromal and Immune Scores With Gastric Cancer Pathology and Prognosis

**Figure 1:**
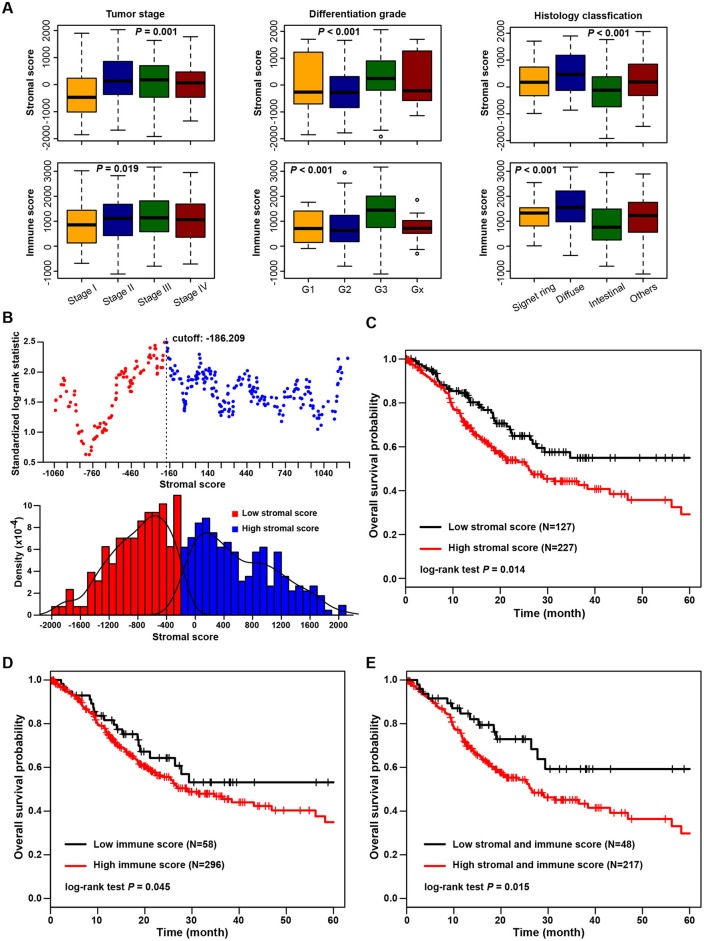
Association of stromal and immune scores with gastric cancer pathology and prognosis. **(A)** Distributions and comparisons of stromal and immune scores among different tumor stages, differentiation grades, and histology classifications. **(B)** An illustration of optimal cutoff identification for stromal score. The upper scatter plot shows the standardized log-rank statistic value for each corresponding expression cutoff. The optimal cutoff (stromal score = −186.209) with the maximum standard log-rank statistic is marked with a vertical dashed line. The lower histogram shows the density distribution for low- and high-stromal score groups divided by the optimal cutoff. **(C)** Kaplan-Meier plot of overall survival for patients with low vs. high stromal scores. **(D)** Kaplan-Meier plot of overall survival for patients with low vs. high immune scores. **(E)** Kaplan-Meier plot of overall survival for patients with simultaneously low stromal and immune scores vs. patients with high stromal and immune scores.

The association of stromal and immune scores with gastric cancer patient pathologic characteristics was examined by comparing the score distributions among different tumor stages, differentiation grades, and histology classifications (Figure 1A). Both stromal and immune scores roughly increased with increasing tumor stage (one-way ANOVA test, *p* = 0.001 for stromal score and *p* = 0.019 for immune score). Significant associations were observed between stromal and immune scores and tumor differentiation grades; tumors with poorer differentiation (G3) yielded higher stromal and immune scores than those differentiated well (G1 and G2) (one-way ANOVA test, both *p* < 0.001). Also, both stromal score and immune score were variously distributed among different histology classifications (one-way ANOVA test, both *p* < 0.001). The diffuse type of tumors had the highest stromal and immune scores, and the intestinal type yielded the lowest scores.

The association of stromal and immune scores with gastric cancer prognosis was evaluated by dividing patients optimally into two groups based on their scores by using standardized log-rank statistics (see Methods for details). An illustration of optimal cutoff identification for stromal score is shown in Figure 1B. Patients with a low stromal score yielded better overall survival than those with a high stromal score (log-rank test *p* = 0.014) (Figure 1C). Similarly, patients with a low immune score had significantly longer mean overall survival time than those with a high score (log-rank test *p* = 0.045) (Figure 1D). Stromal and immune scores were further combined as a new panel, whereby patients with both a high stromal score and a high immune score were compared with those with both a low stromal and a low immune score (Figure 1E). Patients with low scores were found to have significantly better survival than those with high scores (log-rank test *p* = 0.015).

### Comparison of Gene Expression Profile by Immune and Stromal Scores in Gastric Cancer

**Figure 2:**
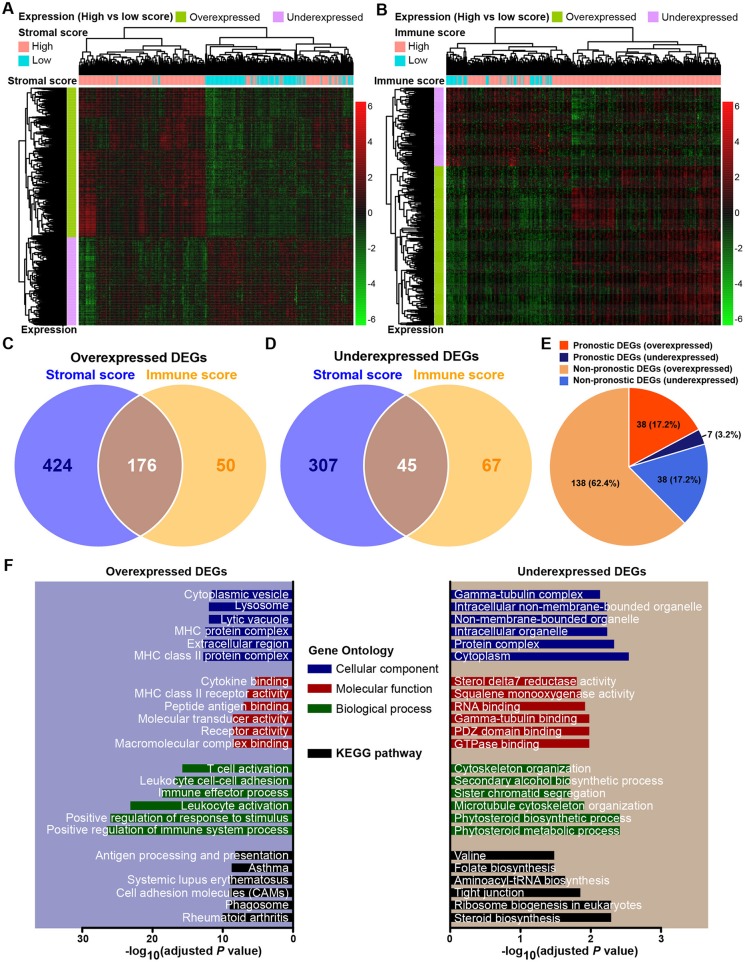
Expression profiles and biological functions of stromal and immune score-related DEGs. **(A,B)** Heatmaps showing expression profiles for stromal score- and immune score-related DEGs with unsupervised hierarchical clustering analyses, using the complete linkage method to measure distances between clusters. **(C)** Overlap of stromal score- and immune score-related overexpressed DEGs. **(D)** Overlap of stromal score- and immune score-related underexpressed DEGs. **(E)** Distribution of prognostic and non-prognostic DEGs among all DEGs. **(F)** Top six Gene Ontology terms and KEGG pathways enriched by the overexpressed and underexpressed DEGs. *P-*values were adjusted by false discovery rate.

The expression profiles of gastric cancer patients with a high immune (or stromal) score were compared to those with a low score to identify immune (or stromal) score-related DEGs. A total of 952 DEGs were identified to be stromal score-related DEGs. Among them, 600 genes were overexpressed (log_2_FC > 4, *p* < 0.0001, adjusted by FDR), and 352 genes were underexpressed (log_2_FC > −4, *p* < 0.0001, adjusted by FDR). For comparison between the high- and low-immune score groups, a total of 338 DEGs were identified as immune score-related DEGs, including 226 overexpressed (log_2_FC > 4, *p* < 0.0001, adjusted by FDR) and 112 underexpressed (log_2_FC > −4, *p* < 0.0001, adjusted by FDR). The expression profiles of stromal and immune score-related DEGs are visualized, respectively on the heatmaps (Figures 2A,B). Unsupervised hierarchical clustering analyses showed the identified DEGs to have distinct expression patterns among analyzed gastric cancer patients, and these DEGs could effectively distinguish patients with high and low scores. There were 176 shared DEGs overexpressed in both the stromal score and immune score groups (Figure 2C), and a total of 45 common DEGs were found to be underexpressed in both the stromal score and immune score groups (Figure 2D). Biological function enrichment analyses found that the overexpressed DEGs were related to immune-related proteins and organelles, such as MHC proteins, and played roles in immune cell and immune system process activation, while the underexpressed DEGs could take part in the tubulin complex, cytoskeleton organization, and tight junctions (Figure 2F).

### Identification of Prognostic DEGs in Gastric Cancer

**Figure 3:**
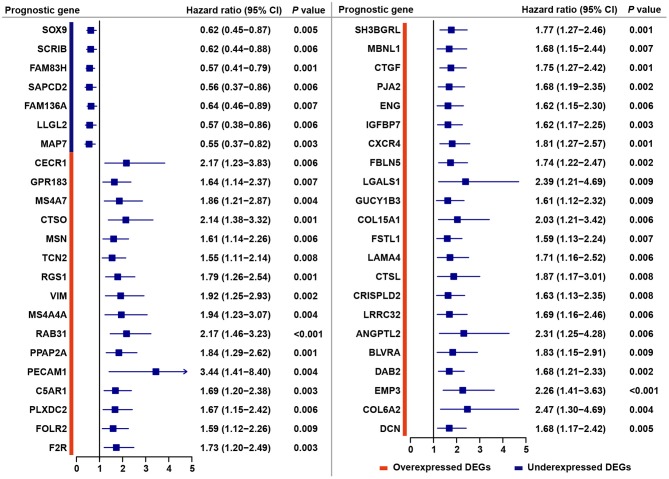
Forest plot of hazard ratios for 45 stromal-immune score-related prognostic DEGs. Hazard ratios and corresponding 95% confidence intervals were estimated by using the Cox proportional hazard regression model.

Through a predefined strategy (see Methods for details), prognostic DEGs that were significantly associated with favorable or unfavorable survival outcomes of gastric cancer patients were identified from the above 221 stromal-immune score-related DEGs, which comprise 176 overexpressed DEGs and 45 underexpressed DEGs shared in both the stromal and immune scores groups. A total of 38 (17.2%) prognostic genes were identified among the 176 overexpressed DEGs, and seven (3.2%) prognostic genes were identified among the 45 underexpressed DEGs (Figure 2E). The prognostic effects of these 45 prognostic DEGs are presented in a forest plot (Figure 3). Interestingly, among these prognostic DEGs, those that were overexpressed were consistently associated with favorable survival outcomes, and those that were underexpressed were in association with unfavorable outcomes.

### Stromal-Immune Score-Based Gene Signature and Risk Stratification Model for Gastric Cancer

**Figure 4:**
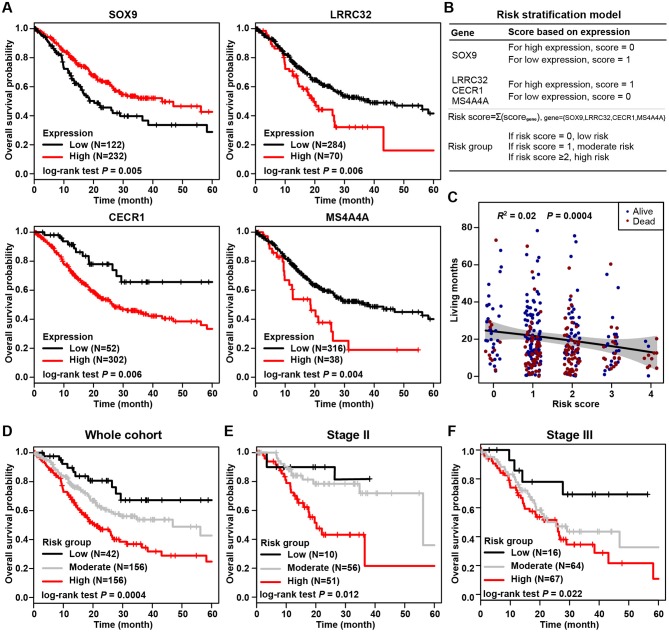
Stromal-immune score-based gene signature and risk stratification model. **(A)** Kaplan-Meier plots of overall survival for patients grouped by expression of four signature genes, SOX9, LRRC32, CECR1, and MS4A4A. **(B)** Algorithm for risk stratification model based on the expression of four stromal-immune score-related signature genes. **(C)** Correlation between risk score and gastric cancer patient living months. **(D)** Kaplan-Meier plot of overall survival for all patients according to risk group. **(E)** Kaplan-Meier plot of overall survival for patients with stage II gastric cancer according to risk group. **(F)** Kaplan-Meier plot of overall survival for patients with stage III gastric cancer according to risk group.

The most prognostic gene signature was then identified from the above 45 prognostic DEGs. By using a robust partial likelihood-based Cox proportional hazard regression survival model (3-fold cross-validation with 1,000 iterations, see Methods for details), a series of models were generated. Four prognostic DEGs, namely SOX9, LRRC32, CECR1, and MS4A4A, were identified as the optimal signature genes based on the smallest values for the negative log-likelihood and AIC statistics (Table 2). Among these four signature genes, SOX9 was a significantly favorable prognostic gene, and LRRC32, CECR1, and MS4A4A were significantly associated with unfavorable survival outcomes (Figure 4A). The prognosis stratification ability of the above four signature genes was further compared with two previously reported signatures, the CXCR family (21) and a cell cycle-related signature (22) (see Methods for details). Better prognostication efficacy was observed for the current stromal-immune score-based gene signatures, which was indicated by a smaller value of the −2log likelihood statistic calculated by Cox regression analyses (Table 3). Based on the four signature genes, a unique individual-level score was developed for risk stratification (Figure 4B). The risk score was a combination of all of the scores that were imputed from the expression of each signature gene (see Method for details). A higher risk score was found to be significantly associated with a shorter survival time, and patient living months gradually decreased with an increasing risk score (*R*^2^ = 0.02, *p* = 0.0004) (Figure 4C). Three risk groups were finally stratified out according to the risk score (see Methods for details). By using this three-grade risk stratification model, the whole cohort was classified into three groups (i.e., low-, moderate-, and high-risk) yielding incremental survival outcomes (log-rank test *p* = 0.0004) (Figure 4D). The risk stratification model was further validated in two subgroups of patients with stage II and stage III gastric cancer, respectively (Figures 4E,F). Similarly to the whole cohort, for both the stage II and stage III disease groups, patients were stratified into three groups with significantly distinct prognosis, and it was found that the higher the risk was, the poorer the patient survival outcome (stratified log-rank test, *p* = 0.012 and *p* = 0.022 for stage II and stage III, respectively).

**Table 2:** Prognostic gene signature selection based on a robust likelihood-based survival model.

**Gene symbol**	**Negative log-likelihood**	**AIC**	**Forward selection**
SOX9	755.69	1513.38	Y
LRRC32	753.85	1511.69	Y
CECR1	753.79	1513.58	Y
MS4A4A	749.62	1507.25	Y
C5AR1	749.48	1508.95	–
DCN	749.24	1510.48	–
IGFBP7	748.95	1511.90	–
LGALS1	748.93	1513.86	–
LLGL2	748.72	1515.44	–
PLXDC2	747.33	1514.65	–
MS4A7	747.10	1516.21	–
TCN2	747.06	1518.13	–
CTSL	746.95	1519.90	–
FAM136A	746.06	1520.12	–
MSN	745.95	1521.90	–

*AIC, Akaike Information Criterion; a smaller statistic value indicates better predictive ability*.

**Table 3:** Comparison of prognosis efficacy of the current signature with two previously developed signatures.

**Signature**	**TCGA**		**GSE84437**		**GSE62254**	
	**−2log likelihood**	***p***	**−2log likelihood**	***p***	**−2log likelihood**	***p***
Current signature	1495.88	<0.001	1512.01	0.002	1507.33	<0.001
CXCR family signature (21)	1685.32	0.005	1683.11	0.004	1502.02	<0.001
Cell cycle-related signature (22)	1501.62	<0.001	1543.96	0.002	1511.03	<0.001

*The statistics of −2log likelihood were estimated by Cox proportional hazard regression analysis*.

### Stromal-Immune Score-Based Risk Group and Clinicopathologic Characteristics in Gastric Cancer

**Figure 5:**
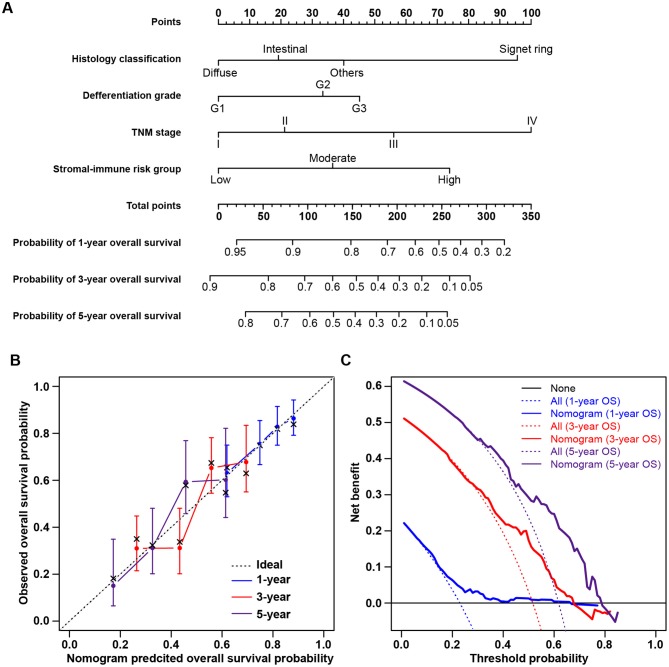
Nomogram for predicting overall survival of gastric cancer patients and decision curve analysis. **(A)** Nomogram to predict 1-, 3-, and 5-years overall survival probability by integrating the stromal-immune score-based risk group and clinicopathologic risk factors. **(B)** Plot depicting the calibration of the nomogram in terms of the agreement between predicted and observed outcomes. Nomogram performance is shown by the plot relative to the dotted line, which represents perfect prediction. **(C)** Decision curve analysis of the nomogram. None: assume an event will occur in no patients (horizontal solid line); All: assume an event will occur in all patients (dash line). The graph shows the expected net benefits based on the nomogram prediction at different threshold probabilities.

The clinicopathological characteristics of patients in the low-, moderate-, and high-risk groups are listed in Table 1. Gender, race, age, and tumor location were comparable among the three risk groups, without significant differences. The high-risk group tended to include more patients with tumors of the signet ring cell and diffuse types (Fisher's exact test, *p* = 0.002) and with poor differentiation (Fisher's exact test, *p* = 0.001). More patients with advanced-stage tumors were included in higher-risk groups, and more early-stage tumors were in lower-risk groups (chi-square test, *p* = 0.044). Consistent with the abovementioned findings, patients in higher-risk groups yielded higher stromal scores (chi-square test, *p* < 0.001) and immune scores (chi-square test, *p* < 0.001). Univariate survival analysis showed that stromal score, immune score, and tumor stage were significant risk factors for overall survival. A multivariate Cox regression model was applied to evaluate the prognostic value of the stromal-immune score-based risk stratification model (Table 4). After adjusting the variables of gender, race, age, tumor location, histology classification, and differentiation grade, the risk group was identified as a significant prognostic factor (adjusted *p* = 0.018), being similar to and independent of tumor stage (adjusted *p* = 0.020). This indicated the potential of the stromal-immune score-based gene signature to be a prognosis stratification tool in gastric cancer. A nomogram for overall survival prediction was further built by integrating the stromal-immune score-based risk group and clinicopathologic risk factors (Figure 5A). The calibration plot showed that the nomogram performed well-compared against the performance of an ideal model (Figure 5B). Decision curve analysis was performed to quantify the clinical usefulness of the nomogram. For 1-, 3-, and 5-years overall survival probability, the decision curve showed that the nomogram provided better net benefits than the alternative options (Figure 5C).

**Table 4:** Multivariate Cox regression analyses of risk factors for overall survival.

	**Adjusted hazard ratio^†^**	**95% confidence interval**	**Adjusted *p***
Tumor stage			0.020
II vs. I	1.65	0.75–3.62	
III vs. I	2.20	1.03–4.68	
IV vs. I	4.12	1.58–10.77	
Risk group			0.018
Moderate vs. Low	1.44	0.67–3.10	
High vs. Low	2.32	1.10–4.90	

† *Adjusted variables included gender, race, age, tumor location, histology classification, and differentiation grade*.

### Validation of Stromal-Immune Score-Based Risk Stratification Model in Two External Cohorts

**Figure 6:**
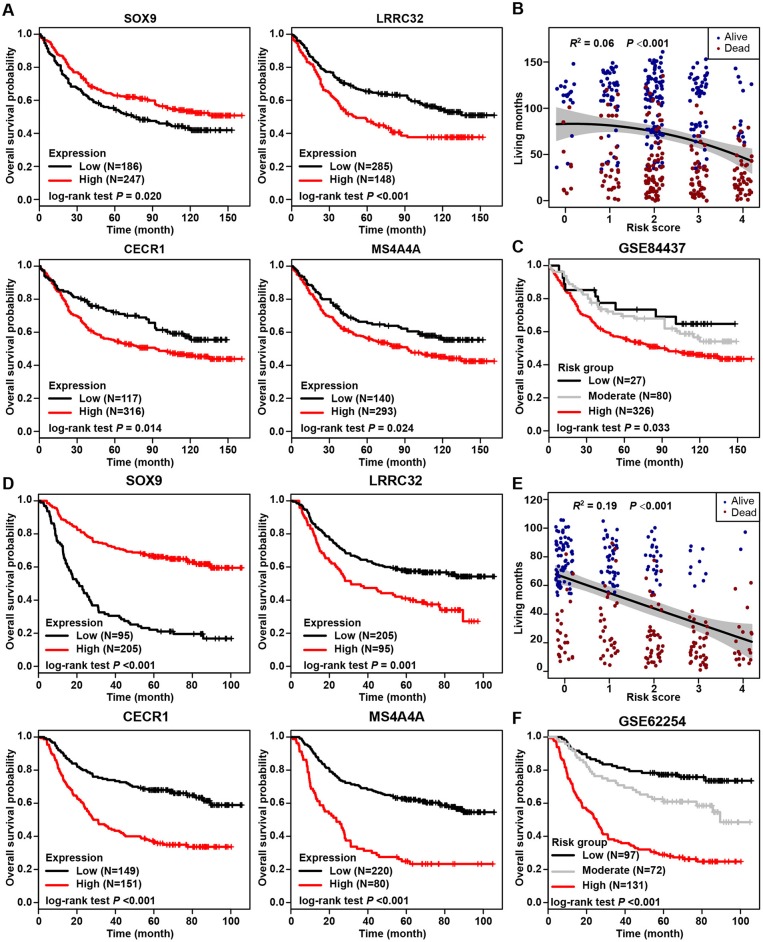
Validation of stromal-immune score-based risk stratification model in two independent cohorts. **(A)** Kaplan-Meier plots of overall survival for patients from GEO Series GSE84437, grouped by expression of four signature genes, SOX9, LRRC32, CECR1, and MS4A4A. **(B)** Correlation between risk scores and living months for patients from GEO Series GSE84437. **(C)** Kaplan-Meier plot of overall survival according to risk group for patients from GEO Series GSE84437. **(D)** Kaplan-Meier plots of overall survival for patients from GEO Series GSE62254 grouped by expression of four signature genes, SOX9, LRRC32, CECR1, and MS4A4A. **(E)** Correlation between risk score and living months for patients from GEO Series GSE62254. **(F)** Kaplan-Meier plot of overall survival according to risk group for patients from GEO Series GSE62254.

The stromal-immune score-based gene signature and risk stratification model were further validated by two independent datasets from the GEO database (Series GSE84437 and GSE62254, see Methods for details). Among 433 patients from Series GSE84437, high expression of SOX9 was significantly associated with favorable overall survival (log-rank test, *p* = 0.020), and LRRC32, CECR1, and MS4A4A were observed as unfavorable prognostic genes (*p* < 0.001, *p* = 0.014, and *p* = 0.024, respectively) (Figure 6A), consistent with the results for the TCGA cohort. The risk scores calculated from these four signature genes also similarly exhibited a significantly negative correlation with patient living months (*R*^2^ = 0.06, *p* < 0.0001) (Figure 6B). By applying the current three-grade stratification model, similar in the TCGA cohort, patients in this validation cohort were classified into three groups with significantly incremental survival outcomes (log-rank test, *p* = 0.033) (Figure 6C). These findings were further validated in another independent cohort containing 300 patients (GSE62254), and similar results were found (Figures 6D–F).

## Discussion

Prognosis prediction and risk stratification for gastric cancer patients still remain challenging for clinicians and investigators. Through a specific view of the microenvironment, this study provides a stromal-immune score-based gene signature to help answer this important clinical question. The stromal and immune scores for tumor tissue were found to be significantly associated with the clinicopathologic characteristics of the tumor (e.g., stage, differentiation grade, and histology classification) and the patient's prognosis. Using a specific analytic framework for transcriptomic data and survival information, this study identified a set of stromal-immune score-related prognostic DEGs and developed a stromal-immune score-based gene signature as a prognosis stratification tool in gastric cancer. Comparisons with two previously reported signatures and validations in two independent cohorts demonstrated promisingly prognostic value and stratifying ability for this tool. This represents a new insight to improve discussions on prognosis prediction and patient stratification through consideration of the microenvironment and transcriptomics.

Tumor progression is affected not only by its intrinsic characteristics but also by extrinsic tumor microenvironment cells. Accumulating evidence has elucidated significant roles for the microenvironment in predicting tumor progression and prognosis (12, 25, 26). Immune system accumulation and immune cell infiltration of the tumor microenvironment could have an impact on carcinogenesis and prognosis (12, 25). Thus, from such specific insights, microenvironment characteristics could provide information for the prediction of tumor outcome and patient prognostication. Ti Wen et al. built an immunoscore system based on components of immune cells in tumor tissues that could improve the accuracy of TNM staging for survival prediction and was an essential complement to the AJCC staging system for patients with stage II/III gastric cancer (27). Additionally, it has also been suggested that the combination of immune features in the tumor microenvironment and TNM stages had better prognostic value than TNM staging alone (15). However, these studies took limited notice of stromal components in the tumor microenvironment. In this study, our analysis comprehensively evaluated the infiltration of stromal cells and immune cells by generating two signature scores, i.e., the stromal score and immune score, respectively, and subsequent analytic series demonstrated that both stromal and immune scores were unfavorable factors for survival outcome and could be used to significantly stratify patient prognoses and improve the prediction accuracy of the TNM staging system in gastric cancer.

Several signature genes identified in this study have been reported previously to play important roles in carcinogenesis in multiple types of cancers, including gastric cancer. LRRC32, located in human chromosomal 11q13-14, has frequently been found to be amplified in tumor tissue, and its overexpression was found to promote Foxp3^+^ regulatory T cell activity, which in turn contributed to enhancing cancer progression and metastasis (28). Another identified unfavorable gene, CECR1, was demonstrated to serve a critical function in M2-like macrophages, mediating cross-talk between macrophages and pericytes in glioblastoma via paracrine PDGFB-PDGFRβ signaling, promoting pericyte recruitment and migration and tumor angiogenesis (29). These results were consistent with our findings that high expression of LRRC32 and CECR1 could have an unfavorable impact on patient's outcomes. However, the role of MS4A4A, a novel cell surface marker for M2-like macrophages and plasma cells (30), has not yet been uncovered clearly in carcinogenesis and tumor progression. In this study, MS4A4A was identified as an unfavorable gene associated with poor prognosis in gastric cancer. This could present an underlying target for experimental design in the laboratory for elucidating molecular mechanisms in tumorigenesis and cancer development.

Investigators have reported many potential targets that could play important roles in carcinogenesis and the cancer process and even their detailed mechanism of action. However, translating these efforts and findings in molecular biology into clinic applications is a constant challenge for clinicians and researchers. Integration of molecular and genetic profiles and clinicopathologic characteristics tends to be typical of this field when considering precision prognostication and individualized treatment. Similar to our study, previous studies have made great efforts to incorporate gene expression profiles into the clinic-used TNM staging system to improve staging accuracy and prognostication for gastric cancer. For example, the G-factor, based on the expression of p53 and MMP-7, was found to be a promising factor for predicting the outcome of Stage II/III gastric cancer and possibly to help select the treatment for stage II cancer, thus supplementing the conventional TNM system (31). Ongoing efforts on human genome characterization are making molecular and genetic variations more and more clear; in the meantime, improvements in sequencing techniques make it possible to perform individual prognostication and risk evaluation from transcriptomic profiles in a clinical setting. Thus, the findings in this study linking the genetic profile and characteristics of the tumor environment with a patient's prognosis could potentially have translational value for clinical management in gastric cancer.

There are also several limitations to this study. First, it was a retrospective study based on publicly available databases, and it was difficult to cover the variation in different geographical regions, although two datasets from Asian areas were used for validation. Second, given that the microenvironment could differ in distinct tumor regions, it is appropriate to conduct immune and stromal components evaluation systematically in the core of the tumor and at the invasive margin. However, the transcriptomic profiles used in this study were all derived from a core sample of tumor tissue, making it impossible to take the different tumor regions into consideration. Thus, a well-designed, prospective, international, multicenter clinical trial is awaited to validate our findings further.

## Conclusion

In summary, the microenvironment characteristics in tumor tissue might be associated with tumor progress and patient survival outcome. Stromal-immune score-based gene signatures can be used to predict survival and add prognostic value to the current TNM staging system. This might serve as a prognosis stratification tool for facilitating patient counseling, decision-making regarding individualized adjuvant treatment, and follow-up scheduling.

## Data Availability Statement

Publicly available datasets were analyzed in this study. These data can be found here: TCGA: https://portal.gdc.cancer.gov/; GEO: https://www.ncbi.nlm.nih.gov/geo/.

## Ethics Statement

Ethical review and approval was not required for the study on human participants in accordance with the local legislation and institutional requirements. Written informed consent for participation was not required for this study in accordance with the national legislation and the institutional requirements.

## Author Contributions

HW and YC guarantee the integrity of the manuscript and contributed to the concept and design. HW, XW, and YC contributed to the data collection, data analysis, and interpretation. All authors contributed to writing and revising the manuscript. All authors read and approved the final manuscript.

### Conflict of Interest

The authors declare that the research was conducted in the absence of any commercial or financial relationships that could be construed as a potential conflict of interest.

## Supplementary Material

Supplementary Table 1 Gene signatures for stromal and immune score estimation.

Supplementary Figure 1 Stromal and immune scores and tumor purity distribution for all analyzed patients. Blue, brown, and gray horizontal solid lines show the mean levels of the stromal score, immune score, and tumor purity, respectively.
