# Supplementary figures and images for: Stromal-Immune Score-Based Gene Signature: A Prognosis Stratification Tool in Gastric Cancer
Source: Front Oncol. 2019 Nov 12;9:1212. doi: 10.3389/fonc.2019.01212 (PMC6861210; doi:10.3389/fonc.2019.01212)

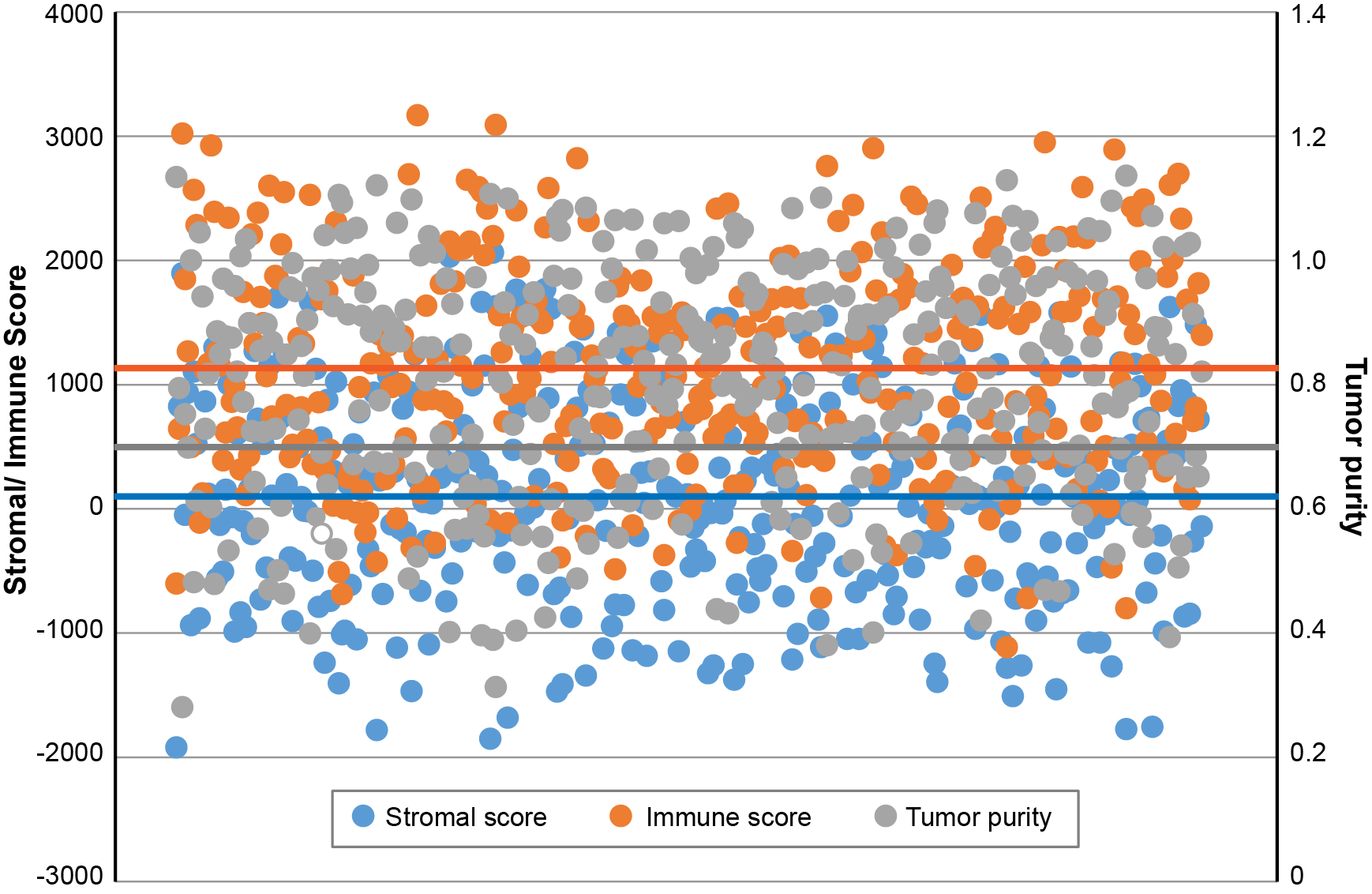

Supplement: Supplementary Figure 1 — Stromal and immune scores and tumor purity distribution for all analyzed patients. Blue, brown, and gray horizontal solid lines show the mean levels of the stromal score, immune score, and tumor purity, respectively. [file Image_1.TIF]
